# Enhancement of Luminescence Efficiency of Y_2_O_3_ Nanophosphor via Core/Shell Structure

**DOI:** 10.3390/nano11061563

**Published:** 2021-06-14

**Authors:** Jae-Young Hyun, Ki-Hyun Kim, Jae-Pil Kim, Won-Bin Im, Kadathala Linganna, Ju-Hyeon Choi

**Affiliations:** 1 Korea Conformity Laboratories, 17-22, Cheomdangwagi-ro 208 beon-gil, Buk-gu, Gwangju 61011, Korea; jyhyun@kcl.re.kr; 2 Intelligent Optical Module Research Center, Korea Photonics Technology Institute, Cheomdanvenchure-ro 108 beon-gil 9, Buk-gu, Gwangju 500-779, Korea; kimkh@kopti.re.kr (K.-H.K.); jpkim@kopti.re.kr (J.-P.K.); 3 Department of Materials Science and Engineering, Hanyang University, 222, Wangsimni-ro, Seongdong-gu, Seoul 04763, Korea; imwonbin@hanyang.ac.kr

**Keywords:** Y_2_O_3_ nanophosphor, core–shell structure, RE ions, luminescence efficiency, quantum yield

## Abstract

We successfully fabricated Y_2_O_3_:RE^3+^ (RE = Eu, Tb, and Dy) core and core–shell nanophosphors by the molten salt method and sol–gel processes with Y_2_O_3_ core size of the order of 100~150 nm. The structural and morphological studies of the RE^3+^-doped Y_2_O_3_ nanophosphors are analyzed by using XRD, SEM and TEM techniques, respectively. The concentration and annealing temperature dependent structural and luminescence characteristics were studied for Y_2_O_3_:RE^3+^ core and core–shell nanophosphors. It is observed that the XRD peaks became narrower as annealing temperature increased in the core–shell nanophosphor. This indicates that annealing at higher temperature improves the crystallinity which in turn enhances the average crystallite size. The emission intensity and quantum yield of the Eu^3+^-doped Y_2_O_3_ core and core–shell nanoparticles increased significantly when annealing temperature is varied from 450 to 550 °C. No considerable variation was noticed in the case of Y_2_O_3_:Tb^3+^ and Y_2_O_3_:Dy^3+^ core and core–shell nanophosphors.

## 1. Introduction

For the past few decades, nanomaterials have received particular attention for luminescence and photonic device application due to their interesting characteristics including high surface-to-volume ratio and the quantum confinement effect. When compared to bulk materials, these nanosized phosphors exhibit considerable modifications in the structure that include size, morphology, and crystallinity. Thus, in particular, rare earth-doped luminescent nanomaterials have been paid much attention as they play an important role in the luminescent devices such as displays, light emitting diodes, biological assays, and optoelectronics [1,2,3,4]. Rare-earth ions in a nanoscale host material are particularly interesting because of their unique properties such as luminescence in all spectral ranges from UV to IR, narrow emission linewidths, longer lifetime, and high quantum efficiency. Of the different sesquioxides, yttrium oxide (Y_2_O_3_) has been studied widely as a host material for rare earth ion doping in photonics and optoelectronics because of its thermo-mechanical properties, optical transparency (0.2–8 μm), and thermodynamically stable crystal structures. In addition, Y_2_O_3_ has a relatively low annealing temperature (400 °C), high refractive index (~1.8), and low maximum phonon energy (380 cm^−1^), which make it a very promising host material for the production of efficient luminescent media, as well as for infrared ceramics [5]. Moreover, Y_2_O_3_ can be doped with different types of RE ions to produce strong luminescence over a wide range of wavelengths due to similarities in the atomic radii, crystal structure, and lattice constant of rare earth ions [6,7]. Among the rare earth ions, the Eu^3+^, Tb^3+^ and Dy^3+^ are particularly interesting for display device applications as they emit the luminescence in the visible region of red, green, and blue arising from the 4f–4f transitions, respectively.

In order to enhance the luminescence efficiency, it is necessary to control the phosphor size and crystallinity. In view of the above importance, numerous studies have been focused on different methods to synthesize nanocrystalline phosphor particles with different size and morphology, such as solid state reaction, spray pyrolysis, co-precipitation, sol–gel, solution combustion, hydrothermal, etc. Of the wet chemical methods, the molten salt synthesis technique has been used to produce single-phase nanoparticles at low processing temperature with a short duration reaction time and less residual filths. It is found that the rare earth-doped phosphors have shown a decrease in luminescence as the size of these particles changes to the nanoscale from the microscale due to the presence of parasitic surface quenching sites or partial diffusion of rare earth ions in host lattices, high rare earth dopant concentrations, and energy transfer to adjacent ions, that can quench the luminescence of rare earth ions in nanoparticles [8].

**Figure 1:**
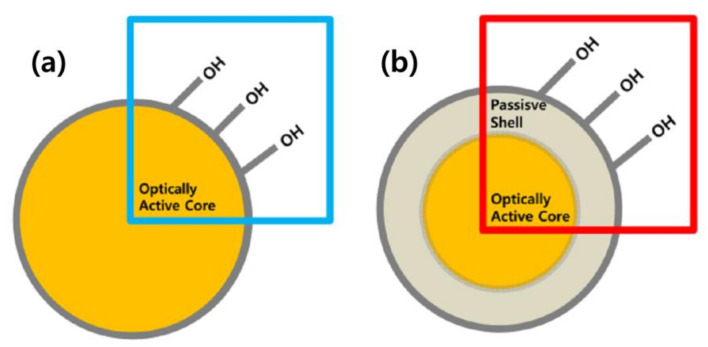
Schematic diagram of the structure of RE-doped (**a**) active core and (**b**) active core@passive shell.

Therefore, it has been demonstrated that the passive shell layer around the core can reduce the surface OH^−^ groups’ absorption and undesirable energy transfer between dopant ions, resulting increase in the luminescence properties [9,10]. The structure of the RE-doped active core as shown in the schematic diagram (see Figure 1a). As can be seen from Figure 1a, the optically active core directly interacts with the surface hydroxyl groups that can quench the luminescence intensity and lifetime of active core. A.K. Parchur et al. [9] and Dorman et al. [10] reported that the effect of the surface hydroxyl groups could be controlled by developing a passive shell layer around the core. The passive shell coated RE-doped active core is shown in the schematic diagram (Figure 1b). The core–shell nanostructures are used to increase the luminescence efficiency by removing the surface quenching sites. The purpose of the passive shell is to increase the distance between the surface quenching site and the doped ions in the core.

Thus, in the present work, RE^3+^ (Eu, Tb, Dy)-doped Y_2_O_3_ nanophosphors with passive shells were fabricated by the molten synthesis method and sol–gel processes and their structural, morphological and luminescence properties studied with core–shell modification for the enhancement of luminescence efficiency.

## 2. Materials and Methods

### 2.1. Fabricated RE-Doped Y_2_O_3_ Core by the Molten Salt Method

The RE (Eu, Tb and Dy)-doped Y_2_O_3_ core nanoparticles were synthesized by molten salt synthesis using analytical grade Y(NO_3_)_3_ and RE(NO_3_)_3_ raw materials with purity of 99.9%. The RE-doped Y_2_O_3_ core nanoparticles were prepared according to the formula (Y_100-x_RE_x_)_2_O_3_ (x = 0.05, 0.10, 0.15, 0.20 for Eu, Tb and Dy). In the molten salt synthesis process, the RE raw materials were mixed with a 1.7:1 mole ratio of NaNO_3_ and KNO_3_ for 10 min, forming a well-mixed powder. Then, the mixture was taken into a covered alumina crucible and heated in an electrical furnace at different temperatures of 450, 500, and 550 °C for 3 h. Then, the powders were cooled down to room temperature. The obtained powder was washed with deionized water until the salt solution was dilute enough to avoid crystallization of the supernatant. The remaining powders were dried at 100 °C overnight.

### 2.2. Y_2_O_3_ Passive Shell Coated Core

Deposition of the RE_2_O_3_ was carried out with the creation of a solution containing 5 mM urea (Sigma Aldrich, Seoul, Korea) and 0.1 mM RECl_3_ (Alfa Aesar 99.9%, Incheon, Korea), depending on the desired thickness. The thickness of the shell layer was controlled by varying the ratio (χ) between the mass of the RECl_3_ precursor and the mass of Y_2_O_3_ core particles. After the urea and RE salts were dissolved, 0.1 g of the Y_2_O_3_ was suspended in the solution and the liquid was sonicated for 30 min, to break apart agglomerates for complete mixing. The solution was heated at 80 °C for 4 h to promote the shell deposition. The resulting suspension was centrifuged and the resulting supernatant solutions were discarded. Afterwards, the particles were dried at 100 °C overnight. Finally, the powder was annealed at 750 °C for 3 h to remove any urea and to crystallize the RE_2_O_3_ shell.

### 2.3. Characterization Techniques

The structural analysis of RE-doped Y_2_O_3_ core nanophosphors was carried out using an X-ray diffractometer (PANalytical, Almelo, Netherlands) with Cu Kα radiation (*λ* = 1.54 Å) as the source in the range of 10° to 80° at a step rate of 0.2°/min for crystalline phase identification. The passive shell layer structure was not analyzed using the XRD technique because of its low sensitivity as well as diffractions peaks overlapping in the range of 0° to 60°. The scanning electron microscope (SEM, Hitachi, S-4700, Houghton, Michigan, USA) was used for the identification of size of the synthesized RE-doped Y_2_O_3_ core and the Y_2_O_3_ passive shell coating thickness was confirmed using a transmission electron microscope (TEM, FEI TECNAI, F20UT, Hillsboro, Oregon, USA). The luminescence spectra were measured for the RE-doped core and core–shell nanoparticles using a spectrofluorimeter (Horiba Jobin Yvon Fluorolog3, Irvine, California, USA). The quantum yield measurements were carried out using an absolute photoluminescence quantum yields measurement system (Horiba Jobin Yvon Fluorolog3, Irvine, California, USA).

## 3. Results and Discussion

**Figure 2:**
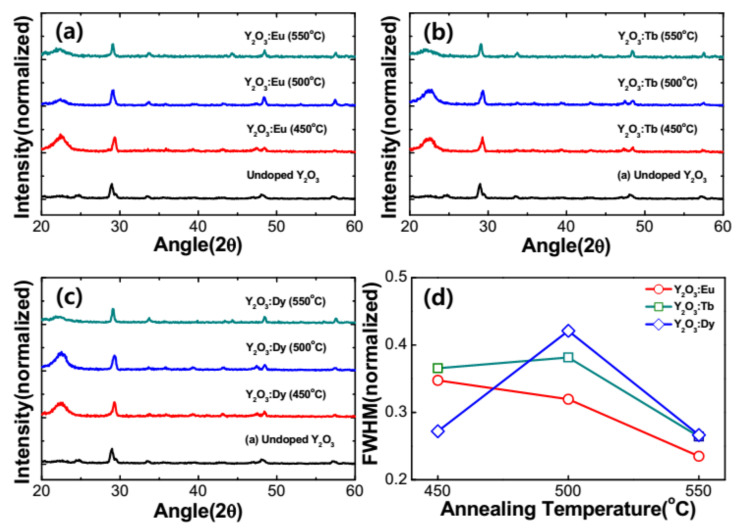
XRD patterns of the RE-doped Y_2_O_3_ for different annealing temperatures at 450, 500, 550 °C. (**a**) 0.05 mM of Eu^3+^-doped Y_2_O_3_ core, (**b**) 0.05 mM of Tb^3+^-doped Y_2_O_3_ core and (**c**) 0.05 mM of Dy^3+^-doped Y_2_O_3_ core, and (**d**) FWHM of RE (Eu, Tb and Dy)-doped Y_2_O_3_ at 450, 500, 550 °C.

Figure 2a–c show the diffraction patterns of the as-prepared and RE^3+^-doped Y_2_O_3_ nanophosphors annealed at different temperatures and were obtained by X-ray diffractometer under the same experimental conditions. The XRD spectra showed a distinctive peaks and clearly indicate crystalline nature even for the as-prepared Y_2_O_3_ sample. The observed diffraction peaks are indexed to (211), (222), (400), (440), and (622) of cubic Y_2_O_3_ and matched to the JCPDS card no. 41–1105. This indicated that the as-prepared and RE^3+^-doped nanoparticles have the characteristic Y_2_O_3_ cubic structure with space group of *Ia3.* A strong peak at 2θ = 29.5° was observed, attributed to the plane (222). Similar behavior has been reported elsewhere [11,12,13,14,15]. An annealing process was performed for the RE^3+^-doped nanophosphors in order to disperse the RE ions into the lattice and in turn to increase the total luminescence of the nanoparticles. The samples were annealed in the range from 450 to 550 °C for 3 h. The position of the diffraction peaks was not changed with the applied annealing temperature and confirm the presence of single cubic crystalline structure of RE-doped Y_2_O_3_ nanoparticles. Figure 2d depicts the variation of peak width (FWHM) as a function of annealing temperature varied from 450 to 550 °C in RE^3+^-doped Y_2_O_3_ nanophosphors. It can be observed that the XRD peaks become narrower on annealing the samples at higher temperatures. Based on the narrowing of the diffraction peaks, it can be concluded that the size of the crystallites increases. An indication of crystallinity increase could be flattening of the amorphous hump (if present) or possibly lowering of the baseline. The crystallize size (D) of RE^3+^-doped nanophosphors can be estimated from the well-known Debye–Scherrer’s equation shown below [16],

$$D=\frac{0.9\lambda }{\beta \cos\theta }$$

where λ is the wavelength of the X-ray source (1.5406 Å), β is the full width at half maximum of intense diffraction peak (222) in the XRD pattern, and θ is the Bragg diffraction angle. The crystallite size of the RE^3+^-doped nanophosphors annealed at different temperatures was calculated using the Scherrer’s equation and tabulated in Table 1. It was noted that the crystallite size of the nanophosphors increased as the annealing temperature increased from 450 °C to 550 °C. The crystallite size of the present nanophosphors calculated from the Scherrer’s equation was compared to the crystallite size of the RE^3+^-doped nanophosphors determined from the Hall–Williamsons equation as shown in Table 1.

**Figure 3:**
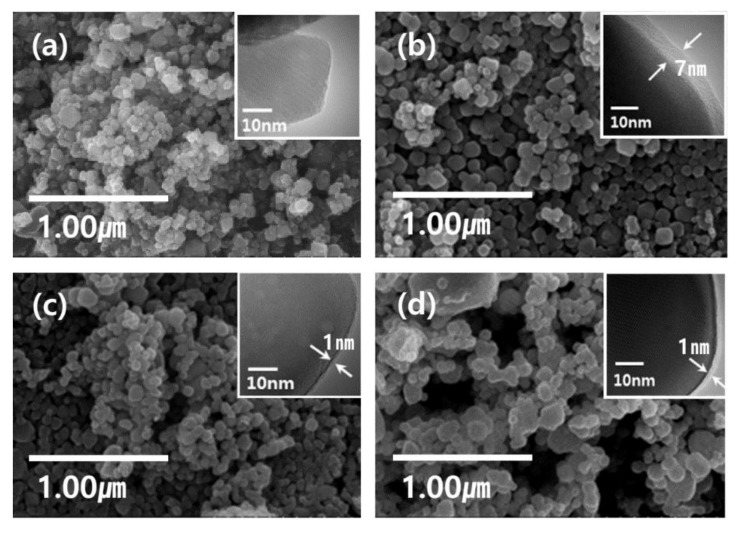
SEM and TEM images (**a**) Y_2_O_3_ NP core sintered at 550℃ for 3 h, (**b**) Eu-doped Y_2_O_3_ NP active core with a passive Y_2_O_3_ shell, (**c**) Tb-doped Y_2_O_3_ NP active core with a passive Y_2_O_3_ shell, (**d**) Dy-doped Y_2_O_3_ NP active core with a passive Y_2_O_3_ shell. The inset TEM images show the coating thickness of each sample.

The morphology of the synthesized undoped and RE^3+^-doped nanophosphors was analyzed by scanning electron microscope (SEM) and transmission electron microscope (TEM) techniques. Figure 3a–d show the SEM images of undoped Y_2_O_3_ and RE^3+^-Y_2_O_3_ (RE = Eu, Tb, and Dy) nanophosphors. It is noticed that the resultant nanoparticles are in a spherical shape and uniform with an average size of approximately 100~150 nm. The inset of Figure 3a–d shows the TEM images of the undoped Y_2_O_3_ and Y_2_O_3_:RE^3+^ (RE = Eu, Tb, and Dy) nanophosphors. The change in mass ratio of the YCl_3_ raw material and Y_2_O_3_ core can control the shell layer thickness deposited around the core. TEM images confirmed the thickness of the core–shell nanoparticles. It is found that the surface of Y_2_O_3_ nanoparticles have irregular shell structures. It is worth mentioning that the exact thickness of the shell layer is difficult to determine from the TEM images due to slight contrast at core–shell interface.

**Figure 4:**
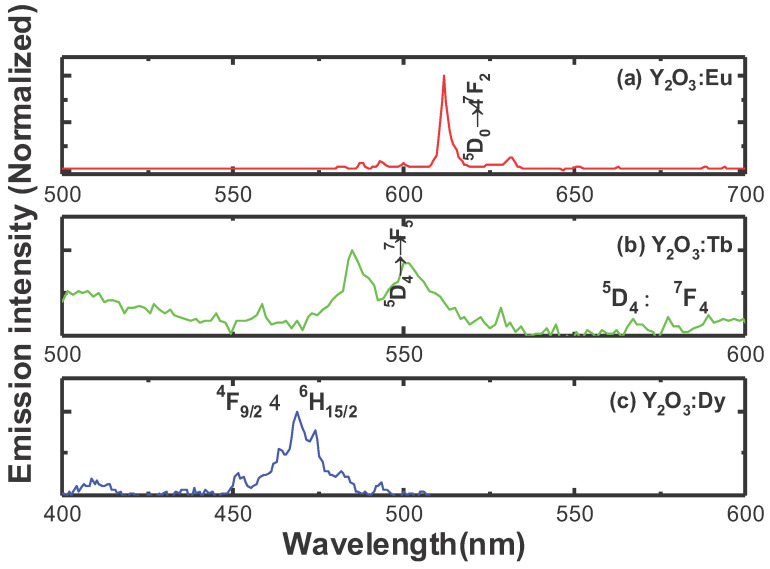
Luminescence spectra of **(a)** 0.05 mMol% Eu:Y_2_O_3,_ **(b)** 0.05 mMol% Tb:Y_2_O_3,,_ and **(c)** 0.05 mMol% Dy:Y_2_O_3_ nanophosphors.

Figure 4a shows the luminescence spectra of 0.05 mMol%Eu^3+^:Y_2_O_3_ nanophosphor, obtained under 254 nm excitation, respectively. As can be seen from the figure, the luminescence spectra exhibited emission lying between 580 nm and 700 nm originating from the ^5^D_0_ → ^7^Fj (j = 0–3) transitions of the Eu^3+^ ion. Among the emission peaks, the strongest peak at 611 nm and a less intense peak at 630 nm attributed to the ^5^D_0_→^7^F_2_ electric-dipole hypersensitive transition of Eu^3+^ ion in the yttrium oxide host and its intensity is sensitive to the environment, while the weak emission peaked at 580 nm corresponds to the ^5^D_0_ → ^7^F_0_ transition. The weak emission in the region 587–600 nm corresponds to the ^5^D_0_ → ^7^F_1_ magnetic-dipole transition of Eu^3+^ ion. Generally, the emission band at 611 nm can show strong emission when Eu^3+^ is located at a lower symmetry (without an inversion center), whereas the emission of magnetic dipole transition at 590 nm is stronger when Eu^3+^ is located at a higher symmetry (with an inversion center). From the emission spectrum of Eu^3+^ ion, it is clearly indicated that the Eu^3+^ ions are situated at lower symmetry as the emission at 611 nm corresponding to the ^5^D_0_→^7^F_2_ transition is dominant. From the emission spectrum in Figure 4a, it is confirmed that Eu^3+^ ions are preferably situated at crystallographic site without inversion center (C_2_) [19]. Figure 4b depicts the emission spectrum of Y_2_O_3_:0.05 mMol% Tb^3+^, obtained under 280 nm excitation. The luminescence spectrum exhibits its strongest peaks at around 545 nm (green emission), and a weak peak centered at 583 nm attributed to the ^5^D_4_→^7^F_5,_ and ^5^D_4_→^7^F_4_ transitions of Tb^3+^ ion, respectively. Figure 4c displays the luminescence spectrum of the Dy^3+^-doped Y_2_O_3_ nanophosphors, obtained under an excitation wavelength of 350 nm. As can be seen from the emission spectrum of the Tb^3+^ ion, it is observed that the Dy^3+^ ion exhibits strong blue emission at around 472 nm attributed to the ^4^F_9/2_ → ^6^H_15/2_ transition of the Dy^3+^ ion (magnetic-dipole transition, insensitive to the local environment).

**Figure 5:**
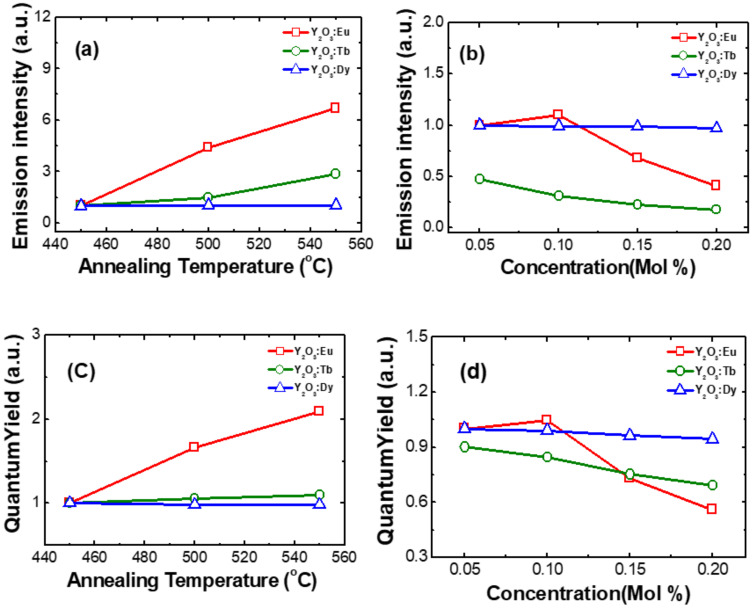
**(a)** Variation of emission intensity with annealing temperature at 450, 500, 550 °C, **(b)** Variation of emission intensity with concentration at 0.05, 0.10, 0.15, 0.20 mMol%, **(c)** Variation of quantum yield with annealing temperature at 450, 500, 550 °C, and **(d)** Variation of quantum yield with concentration at 0.05, 0.10, 0.15, 0.20 mMol% in RE^3+^ (Eu, Tb, Dy)-doped nanophosphors.

Based on the reported literature, it can be noticed that the RE^3+^ ion’s luminescence is often improved by thermal treatment. In this study, the effect of the annealing temperature on the luminescence properties of nanophosphors was investigated. The RE^3+^-doped Y_2_O_3_ nanophosphors were annealed in the temperature range from 450 to 550 °C. Figure 5a shows the variation of the luminescence intensity of RE^3+^-doped Y_2_O_3_ nanophosphor in the dependence of annealing temperature. It was found that the emission intensity of Eu^3+^ ion increased with the increase in annealing temperature due to the improvement of crystallite size in the Y_2_O_3_ nanophosphors. The same trend was observed in Eu^3+^-doped Y_2_O_3_ nanoparticles synthesized by the other methods [11,18,20]. The variation of emission intensity in Tb^3+^-doped Y_2_O_3_ nanophosphors in the dependence of annealing temperature as shown in Figure 5a. The emission intensity of Tb^3+^ ions at the green region increased with the increase in annealing temperature which changed from 450 to 550 °C. The trend of emission intensity in the dependence of annealing temperature could be due to a reduction in surface OH groups and the improvement of crystallite size of the nanophosphors. Figure 5a shows the variation of emission intensity for different annealing temperatures in Dy^3+^-doped Y_2_O_3_ nanophosphors. No considerable variation in the emission intensity for the annealed Dy^3+^-doped nanophosphors. This indicates that the Dy^3+^ environment was not changed with annealing temperature.

The effect of Eu^3+^ ions concentration on luminescence properties in Y_2_O_3_ nanophosphors was investigated as shown in Figure 5b. The emission intensity of Eu^3+^-doped Y_2_O_3_ nanophosphors (at 611 nm) increased with the increase in Eu^3+^ concentration up to 0.1 mol% and then decreased for further increase in Eu^3+^ ion concentration. The optimum Eu doping concentration in Y_2_O_3_ nanophosphor was obtained as 0.1 mol%. The enhanced intensity can be attributed to the increased luminescence active centers, while it decreased for higher Eu doping due to energy transfer through the cross-relaxation mechanism: Eu^3+^ (^5^D_1_) + Eu^3+^ (^7^F_0_) → Eu^3+^ (^5^D_0_) + Eu^3+^ (^7^F_1_) [21]. Figure 5b shows the emission intensity of Tb^3+^-doped Y_2_O_3_ nanophosphor with varying concentration. As the concentration of Tb^3+^ increases, the luminescence intensity decreases in the synthesized nanphosphors. The trend of the Tb^3+^ ion emission intensity with respect to concentration due to the energy transfer through the cross-relaxation mechanism between neighboring Tb^3+^ ions: Tb^3+^ (^5^D_3_) + Tb^3+^ (^7^F_6_) → Tb^3+^ (^5^D_4_) + Tb^3+^ (^7^F_0_). The intensity of the Dy^3+^ characteristic transition was not changed considerably with respect to the Dy^3+^ concentration varied from 0.05 to 0.2 mol% as shown in Figure 5b.

The photoluminescence QY of RE^3+^-doped Y_2_O_3_ nanophosphors was analyzed for different annealing temperatures and RE^3+^ ion concentrations as shown in Figure 5c,d. In the case of Eu^3+^-doped Y_2_O_3_ nanophosphor, the QY increased when the annealing temperature varied from 450 to 550 °C. When we consider the concentration, QY initially increases up to 0.1 mol% and then decreases after further increase in concentration up to 0.2 mol%. This illustrates the importance of considering different concentrations. The QY of Y_2_O_3_:Tb^3+^ and Y_2_O_3_:Dy^3+^ nanophosphors showed slight variation when annealing temperature and concentration varied as illustrated in Figure 5c,d.

**Figure 6:**
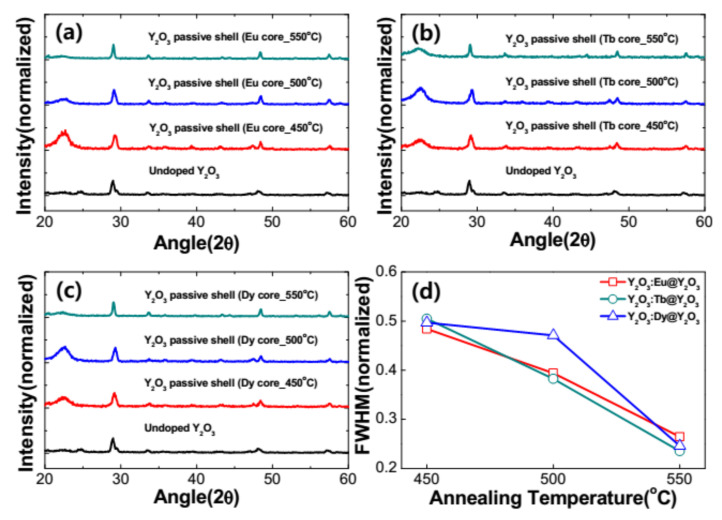
XRD patterns of the RE-doped Y_2_O_3_ core coated with Y_2_O_3_ passive shell via different annealing temperatures at 450, 500, 550 °C. (**a**) An amount of 0.05 mM of Eu^3+^-doped Y_2_O_3_ core with a Y_2_O_3_ passive shell, (**b**) 0.05 mM of Tb^3+^-doped Y_2_O_3_ core with a Y_2_O_3_ passive shell and (**c**) 0.05 mM of Dy^3+^-doped Y_2_O_3_ core with a Y_2_O_3_ passive shell, and (**d**) FWHM of RE (Eu, Tb and Dy)-doped Y_2_O_3_ core with a Y_2_O_3_ passive shell at 450, 500, 550 °C.

Figure 6a–c show the diffraction patterns of the as-prepared and RE^3+^-doped Y_2_O_3_ core with Y_2_O_3_ passive shell nanophosphors annealed in the temperature range from 450 to 550 °C. From the figure, it is clear that the similar XRD patterns are observed for RE^3+^-doped Y_2_O_3_ core with Y_2_O_3_ passive shell nanophosphors when compared to the core nanoparticles. This indicates that the RE^3+^-doped nanoparticles exhibited a Y_2_O_3_ cubic crystal structure even with RE doping, a core–shell structure and annealed at different temperatures. Figure 6d presents the variation of FWHM with respect to the annealing temperature for the RE^3+^-doped Y_2_O_3_ core with Y_2_O_3_ passive shell. It is observed that the FWHM of RE^3+^-doped Y_2_O_3_ core with Y_2_O_3_ passive shell were analogous to the RE^3+^-doped Y_2_O_3_ core nanoparticles. The crystallite size of the nanophosphors was also improved with the core–shell nanostructure.

**Figure 7:**
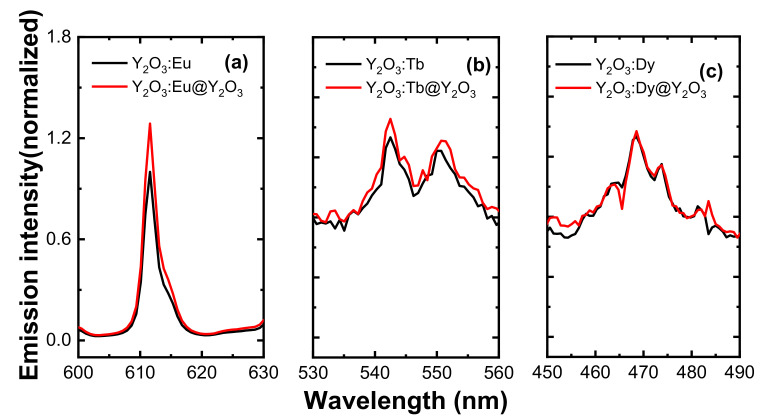
Relative luminescence spectra of the RE-doped Y_2_O_3_ core and Y_2_O_3_ passive shell-coated Y_2_O_3_:RE core. (**a**) Relative luminescence spectra of 0.10 mM of Eu^3+^-doped Y_2_O_3_ core and Y_2_O_3_:Eu@Y_2_O_3_, (**b**) relative luminescence spectra of 0.05 mM of Tb^3+^-doped Y_2_O_3_ core and Y_2_O_3_:Tb@Y_2_O_3_, (**c**) relative luminescence spectra of 0.05 mM of Dy^3+^-doped Y_2_O_3_ core and Y_2_O_3_:Dy@ Y_2_O_3_. All the core samples were annealed at 550 °C for 3 h and passive shells were annealed at 750 °C for 3 h.

**Figure 8:**
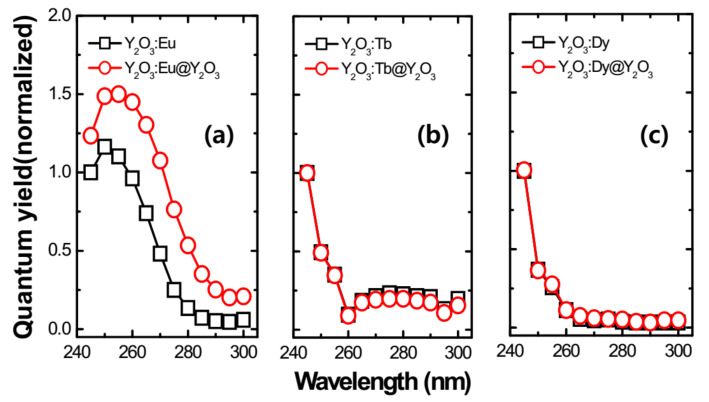
Relative quantum yield spectra of the RE-doped Y_2_O_3_ core and Y_2_O_3_ passive shell-coated Y_2_O_3_:RE core. (**a**) Relative quantum yield of 0.10 mM of Eu^3+^-doped Y_2_O_3_ core and Y_2_O_3_:Eu@Y_2_O_3_, (**b**) relative quantum yield of 0.05 mM of Tb^3+^-doped Y_2_O_3_ core and Y_2_O_3_:Tb@ Y_2_O_3_, (**c**) relative quantum yield of 0.05 mM of Dy^3+^-doped Y_2_O_3_ core and Y_2_O_3_:Dy@ Y_2_O_3_. All the core samples were annealed at 550 °C for 3 h and passive shells were annealed at 750 °C for 3 h.

The 0.10 mMol% of Eu^3+^, 0.05 mMol% of Tb^3+^, and 0.05 mMol% of Dy^3+^-doped Y_2_O_3_ samples were chosen to compare the luminescence properties of RE^3+^-doped Y_2_O_3_ cores and RE^3+^-doped Y_2_O_3_ cores with Y_2_O_3_ passive shells as they exhibited the optimum luminescence intensity. Figure 7 definitively shows that the core–shell phosphors revealed a higher peak intensity as compared to the core nanoparticles due to increase in distance between surface quenching sites and RE^3+^ active ions. After the passive shell coating, the QY was also measured for the RE^3+^-doped core–shell nanoparticles along with RE^3+^-doped core nanoparticles (see Figure 8). Variation in emission intensity (Figure 4 and Figure 5a,b) is qualitative. In order to perform quantitative measurement, the photoluminescence quantum yield (QY) (based on integrated sphere) was carried out for the synthesized RE^3+^-doped nanophosphors with a core–shell structure. The photoluminescence QY is defined as the ratio of number of photons emitted to the number of photons absorbed. All of the reflected and emitted light can be collected where the concern regarding the angular dependence of the photoluminescence emission is no longer needed by implementing integrating sphere into photoluminescence QY measurements. Mello et al. [22] developed an equation for the determination of absolute photoluminescence QY as shown below,

$$\Phi_{PL}=\frac{E_{in}\left(\lambda \right)-\left(1-\alpha \right)E_{out}\left(\lambda \right)}{X_{empty}\left(\lambda \right)\alpha }$$

with

$$\alpha =\frac{X_{out}\left(\lambda \right)-X_{in}\left(\lambda \right)}{X_{out}\left(\lambda \right)}$$

In the above equations, *E_in_(λ)* and *E_out_(λ)* are the integrated luminescence as a result of direct excitation and secondary excitation of the sample, respectively. The latter emission is due to reflected excitation light from sphere walls hitting the sample. *X_empty_(λ)* is the integrated excitation profile with the empty sphere. α is the sample absorbance. The product of the photoluminescence QY and absorption coefficient reveals the brightness of a photoluminescent nanoparticle of the material. The absorption is an intrinsic characteristic of the material, while photoluminescence QY depends on the architecture of the nanoparticle and its immediate environment. It is widely accepted that core/shell engineering of RE-doped nanophosphors allows researchers to substantially improve the photoluminescence QY of the nanophosphors and subsequently their brightness, by means of an optically undoped passive layer. Safeguarding the emission intensity by a passive layer is attained by spatially separating the optically active core from the structural surface defects at the core interface. It can be seen that the QY increased significantly after shell coating in the Y_2_O_3_:Eu^3+^ nanophosphor. No considerable variation was noticed in the case of Y_2_O_3_:Tb^3+^ and Y_2_O_3_:Dy^3+^ nanophosphors.

## 4. Conclusions

In this work, the luminescence properties of RE^3+^-doped Y_2_O_3_ core and core–shell nanoparticles synthesized by the molten salt synthesis and sol–gel processes were investigated in the dependence of annealing temperature and active ion concentration. The morphology studies of the Y_2_O_3_ core and core–shell nanoparticles confirmed the nanoparticle size as approximately 100–150 nm in diameter with shell layer thickness up to 8 nm. The diffraction patterns of as-prepared, RE-doped core, and core–shell nanoparticles annealed at different temperatures showed a cubic Y_2_O_3_ crystal structure (JCPDS 41-1105). The optimum active ion concentration based on luminescence intensity was found to be 0.10 mMol%, 0.05 mMol%, and 0.05 mMol% for Eu^3+^-, Tb^3+^-, and Dy^3+^-doped nanophosphors, respectively. The luminescence intensity and quantum yield of RE-doped core–shell nanoparticles were compared with the Y_2_O_3_ core nanoparticles. The luminescence intensity and quantum yield of Eu^3+^-doped core nanophosphors enhanced after coating with the Y_2_O_3_ passive shell layer. No considerable variation was noticed in case of Tb^3+^- and Dy^3+^-doped Y_2_O_3_ nanophosphors.

## Figures and Tables

**Table 1:** Structural parameters of the RE^3+^-doped nanophosphors.

Sample	Annealing Temperature (°C)	Crystallite Size, D (nm)		Reference
		Debye–Scherrer	Hall–Williamsons	
Y_2_O_3_:Eu	450	23		Present work
	500	25		
	550	34		
Y_2_O_3_:Tb	450	22		
	500	21		
	550	31		
Y_2_O_3_:Dy	450	30		
	500	19		
	550	30		
Y_2_O_3_:Tb	500		15	[17]
Y_2_O_3_:Eu	500		18	[18]

## Data Availability

Not applicable.
